# Cellulose Nanofiber-Assisted Dispersion of Halloysite Nanotubes via Silane Coupling Agent-Reinforced Starch–PVA Biodegradable Composite Membrane

**DOI:** 10.3390/membranes12020169

**Published:** 2022-01-30

**Authors:** Han Li, Jisheng Yang, Xiaoqiong Feng, Zhiyong Qin

**Affiliations:** School of Resources Environment and Materials, Guangxi University, Nanning 530004, China; lihan_14@163.com (H.L.); yangjisheng@bjfu.edu.cn (J.Y.); xiaoqiongfeng2017@163.com (X.F.)

**Keywords:** halloysite nanotubes, cellulose nanofiber, silane coupling agent, starch–polyvinyl alcohol membrane, properties

## Abstract

HNTs (halloysite nanotubes) are widely used in reinforcing material, often used in material reinforcement and particle loading. However, their easy agglomeration causes them to have great limitations in application. In this work, two kinds of silane coupling agents (KH560 and KH570) were introduced to graft the CNF/HNT (cellulose nanofiber) nanoparticles used to reinforce the starch-polyvinyl alcohol (PVA) composite membranes. The mechanical properties, water resistance properties and thermal performance of the composite membrane were tested. The results showed that the CNF/HNTs nanoparticle system modified by two silane coupling agents enhanced the tensile strength (TS) of the starch–PVA composite membranes by increments of 60.11% and 68.35%, and, in addition, the water resistance of starch–PVA composite membrane improved. The introduction of chemical bonds formed associations and a compact network structure, which increased the thermal stability and the crystallinity of the starch–PVA composite membrane. In the study, we creatively used CNF to disperse HNTs. CNF and HNTs were combined under the action of the silane coupling agent, and then mixed into the starch–PVA membranes matrix to prepare high-performance degradable biological composite membranes.

## 1. Introduction

Plastic packaging materials made of polymer materials with advantages of light weight, stable performance, and being resource-rich have been widely used in many fields. The petroleum-based plastics are largely used in several fields, such as packaging, and have generated relevant environmental problems because of their non-degradability. With the increased awareness of sustainable development and environmental protection, the production of biodegradable materials from renewable resources, such as starch, chitosan, protein, fiber and so on, have attracted considerable attention from researchers and developers [1]. Among all the biopolymer studies, starch is used as the matrix for the research and development of packaging materials [2]. Starch is a kind of green biodegradable polymer that can be obtained from many renewable resources. It is a polysaccharide polymer with easy availability and low cost [3]. These advantages give starch many applications in food, medicine and chemical industries [4,5].

However, it is generally known that inferior physical–mechanical properties of starch composite restrict its wide application [6]. A series of methods have been used to improve the mechanical properties and water barrier properties of starch-based composites, including surface modification [7], chemical cross-linking [8,9], ultrasound modification [10,11], filling of nanoparticles [12], and layer-by-layer structure design [13,14,15]. Adding nanoparticles into the starch composite can effectively enhance its physical and mechanical properties and water resistance properties, and some researchers have performed experiments to investigate nanoparticles, such as nanosilicon dioxide [16], clay [17], zeolite and beidellite [18], nanotubes [19,20], montmorillonite [21], nanoparticles of metals [22], and so on. By adding polypropylene [23], PVA [24,25] and other reinforcing materials into the starch membrane, the physical mechanics of the starch membrane can be improved. PVA is a thermoplastic and biocompatible petroleum-based polymer. It is also one of the rare polymers with a C-C single bond backbone that is fully biodegradable. Fabricating biodegradable starch–PVA composites may be a potential way to address some limitations of the starch [26].

HNT is a silicate compound. The main components are silica and alumina compounds and a small amount of ferrite and calcium oxide impurities. It is a multi-wall nanotube formed by the bending of kaolinite flakes under natural conditions. The outer layer of HNT is silica tetrahedron and the inner layer is alumina octahedron. The inner surface properties are similar to Al_2_O_3_ and the chemical properties are similar to SiO_2_. It is a rigid material with a unique crystal structure, which is an ideal material for preparing polymer composites [27]. HNTs are usually used for surface modifications because of their good biocompatibility and good surface reactivity [28]. Silane grafting can be used to improve the HNT dispersion in polymeric/fluidic materials [29]. HNTs have high crystallinity and are difficult to disperse in water. The CNF obtained by TEMPO oxidation method has good dispersion and stability in the solution. It had become the focus of many research fields because of its high Young’s modulus, dimensional stability and good transparency. The type and amount of nanocellulose composites have some effects on the properties of the composites. Under the same conditions, the tensile strength of CNF is better than that of CNC. Nowadays, some progress has been made in the properties of polymers such as CNF reinforcement, thermoplastic starch and polyvinyl alcohol. Combined with the selective modification of functional groups on the surface of nanofibers, new cellulose derivatives are prepared, which improves the application potential of nanocellulose in a larger field. Moreover, CNF has strong mechanical properties [30], biodegradability, compatibility and a rich-charged group (–COO–) on the surface, so it has excellent adsorption properties of the nanoparticles and prevents the re-aggregation of nanoparticles [31,32,33]. Therefore, the dispersion of nanoparticles in solution is effectively promoted by CNF.

This article attempts to find a simple and effective functionalization method of natural HNTs. In this study, the CNF was used to reduce the agglomeration of HNTs, for which CNF and HNTs were combined under the action of silane coupling agent and then mixed into a starch–PVA membranes matrix to prepare high-performance degradable biological composite membranes.

## 2. Materials and Methods

### 2.1. Materials

Starch was provided by Red Maple Co., Ltd. (Nanning, China). HNTs were acquired from Nanocellulose Technology Research and Development Center (Nanning, China). CNF was donated by Zhongke Guochang Technology Co., Ltd. (Beijing, China). Silane reagents including γ-(2,3-Epoxypropoxy) propyltrimethoxysilane (KH560), 3-propyltrimethoxysilane (KH570), PVA, and acetic acid were purchased from Macklin Chemical Industry Co., Ltd. (Shanghai, China). Glycerol (99% purity) and other chemical reagents of analytical reagent grade were purchased from Beijing Chemical Reagents Company (Beijing, China). All chemicals were used without further purification.

### 2.2. Preparation of KH560 and KH570 Modified CNF/HNTs

Two identical mixtures were prepared: HNTs (1 g) and CNF (1 g) powder were added into an ethanol/water (95:5, *v*/*v*) solution to form a mixture. The pH value of the mixture was adjusted to 4.0 using acetic acid (20%, *w*/*w*) under magnetic stirring. Then, KH560 (2 g) was added dropwise into one of the mixtures and KH570 (2 g) was added dropwise into the other one, after that the mixture was stirred with a mechanical stirrer at 80 °C for 8 h. The resulting mixture was pumped, filtered and washed three times with ethanol/distilled water (95:5, *w*/*w*). Finally, the mixture was dried to constant weight at 80 °C.

### 2.3. Preparation of Starch–PVA Composite Membranes

A membrane without any nanofillers was set as a control sample. Membrane-forming solutions (MFS) with nanofillers and MFS without nanofillers were prepared separately. PVA solutions (5%, *w*/*w*) were prepared by dissolving PVA powder in the deionized water at a weight ratio of 5:95 to form PVA solution, which was maintained in a water bath at 100 °C and continuously stirred. Starch solutions (5%, *w*/*v*) were prepared by dissolving starch powder in distilled water, glycerol (30% *w*/*w* of starch) was added as a plasticizer, and the mixture was stirred at 90 °C for 45 min. Nanofillers (1 g) were mixed with water (99 g) and dissolved into nanofillers solution (1%, *w*/*w*) by ultrasound. Starch–PVA blend solution was prepared by mixing starch and PVA at a 2:1 ratio. Moreover, different types of nanofillers (5% of starch–PVA, *w*/*w*) were added to the solution, followed by stirring for an additional 10 min. All MFS were treated with ultrasonic for 30 min. Membranes were obtained by casting 40 g of the MFS into Teflon-coated plates and drying at 40 °C overnight at ambient relative humidity (RH) of 40%. All the membranes were conditioned in a desiccator (57 ± 2 RH% and 25 ± 2 °C) for 48 h before characterization. The detailed code and formulations of all the composite membranes are described in Table 1.

### 2.4. Characterization

#### 2.4.1. Scanning Electron Microscopy (SEM)

Morphology of the cross-section of the membranes was observed using a SUPRA55 field emission scanning electron microscopy (SEM, ZEISS, Industrial Measurement Technology Co., Ltd., Shanghai, China) operating at an acceleration voltage of 10 kV with 2500× magnification. The samples were sputter-coated with a 10 mm gold layer before observation to prevent electron beam charging.

#### 2.4.2. Fourier Transform Infrared Spectroscopy (FTIR)

Fourier transform infrared spectroscopy (FTIR) was carried out on a Nicolet IS 50 spectrometer (Nicolet 7600 Nico-let Instrument Corporation, Madison, Wisconsin) over the range of 4000~750 cm^−1^, with the assistance of an attenuated total reflectance accessory (ATR) with a diamond crystal.

#### 2.4.3. X-ray Diffraction Test (XRD)

X-ray diffraction (XRD) was performed on a D8 advance diffractometer (MINFLEX600, Science Company Co., Ltd., Nanjing, China) equipped with a Cu Kα radiation source (λ = 0.154 nm) at 40 kV and 40 mA. The diffraction data were collected from 2θ values of 5° to 60° at a scanning speed of 5°/min.

#### 2.4.4. Thermogravimetric Analyzer Test (TGA) and Differential Scanning Calorimetry (DSC)

Thermal stability of membranes was analyzed by thermo-gravimetric analysis (TGA, Q50, TA Instruments, New Castle, DE, USA). Non-isothermal degradation measurements were conducted on a DTG-60(H) TGA device (SHIMADZU, Tokyo, Japan). Tests were running from room temperature to 600 °C at a heating rate of 10 °C/min under a flow of 100 mL/min nitrogen gas.

Differential scanning calorimetry (DSC214, NETCH COMPANY, Shenzhen, China) was used to analyze the glass transition temperature (T_g_), melting temperature (T_m_), enthalpy of fusion and crystallinity of nanocomposites [34]. All specimens were dried in an oven at 105 °C for 12 h prior to conducting the test. All DSC analyses were performed under an inert atmosphere by flowing nitrogen at the rate of 50 mL/min, between −20 and 250 °C and at a scanning rate of 10 °C/min.

#### 2.4.5. Water Resistance Properties

The percentage of moisture content (MC%) and moisture absorption (MA%) values were measured at 20 °C, 101.325 KPa, calculated on the dry weight basis. Square-shaped samples (dimension 1 cm^2^) were weighed and marked as m_0_. Samples were then dried at a desiccator (regulated by P_2_O_5_) for 24 h, and reweighed (m_1_). MC was calculated as Formula (1):

(1) $$MC=\frac{m_{0}-m_{1}}{m_{0}}\times 100\%$$

where m_o_ was the initial weight of the sample (g), m_1_ was the dried mass (g).

Afterwards, samples were placed in a 100% relative humidity (RH) desiccator (regulated by water) for 24 h and reweighed (m_2_). MA was calculated as Formula (2):

(2) $$MA=\frac{m_{1}-m_{2}}{m_{1}}\times 100\%$$

where m_2_ was the final mass of the sample (g).

Eight specimens of each sample were tested to determine water vapor permeability (WVP) [32,33,34,35]. Each sample was cut into φ50 mm round. Then, the sample membranes were coated on the mouth of the permeation cup and the initial mass was recorded. The permeation cup was put into a sealed hygroscopic container (20 ± 2 °C, 80 ± 2% RH) and weighted every hour for a total of 24 h. WVP was calculated as Formula (3):

(3) $$WVP=\frac{\Delta M\times d}{A\times t\times \Delta p}$$

where Δ*M* is the different weighted value of the sample by each hour (g), *d* is the mean thickness of the sample (mm), *A* is the area of the sample (m^2^), *t* is the interval (h), Δ*p* is water vapor pressure difference on both sides of the sample (Pa).

#### 2.4.6. Mechanical Properties

Tensile properties were determined using a tensile testing machine (AG-X Plus, Shimadzu Corporation, Tokyo, Japan) according to ISO527-3:1995 (E). For analysis, each sample was cut into 10 × 80 mm^2^ pieces and stress–strain curves were developed [34]. The thickness of each membrane was measured five times using a digital spiral micrometer (Measuring and Cutting Tool Co., Ltd., Nanning, China) with a sensitivity of 0.001 mm. The average of the five data points were used to calculate the tensile strength (TS) and elongation at break (EB).

#### 2.4.7. Statistical Analysis

Sisvar 5.0 was used for statistical analysis of data by average comparison. The Fisher’s least significant difference and Scott–Knott test were used at 95% confidence level for mechanical and physical properties.

## 3. Results and Discussion

### 3.1. CNF Disperse HNTs

**Figure 1:**
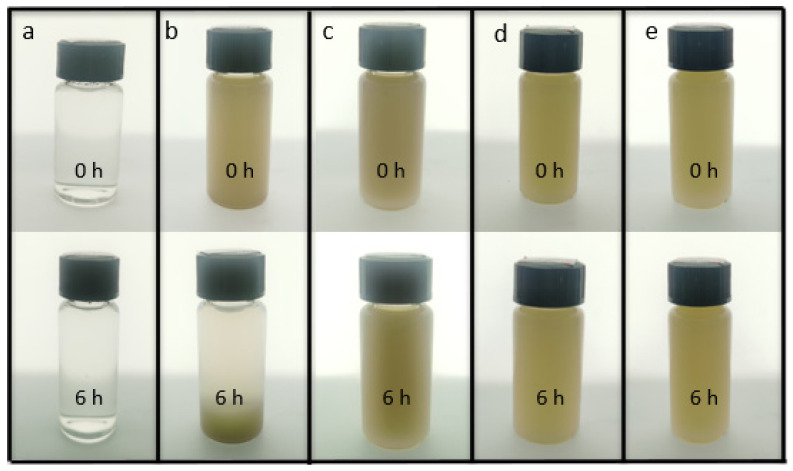
The initial states and states after 6 h of (**a**) CNF; (**b**) HNTs; (**c**) CNF/HNTs; (**d**) HNTs-KH560-CNF; (**e**) HNTs-KH570-CNF nanoparticle solution.

The HNT samples were difficult to disperse in water due to their high crystallinity. Given that CNF could be well dispersed in water, it can be used as a dispersant to improve the dispersion of HNTs. The CNF was connected with HNTs through hydrogen bonds, which made the HNTs more stable and they dispersed better in water. Figure 1 shows the dispersion of sample CNF (a), HNTs (b), CNF/HNTs (c), HNTs-KH560-CNF (d) and HNTs- KH570-CNF (e) in water for 6 h. It can be seen that the solution with only HNTs (Figure 1b) had obvious stratification after six hours. After adding CNF, the system became uniform (Figure 1c). Then, after modification with silane coupling agent, the CNF/HNT nanoparticle system was further cross-linked, thus uniform and stable HNT dispersion could be obtained (Figure 1d,e) [36].

### 3.2. Mechanical Properties Analysis

**Figure 2:**
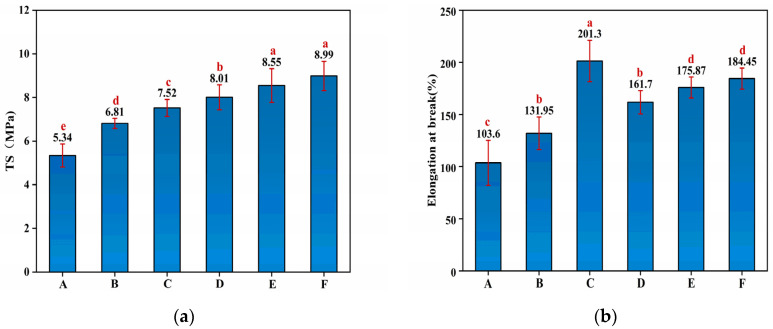
The mechanical properties of (**a**) TS; (**b**) EB of composite membrane samples of starch– (A) PVA; (B) HNTs/starch–PVA; (C) CNF/starch–PVA; (D) HNTs–CNF/starch–PVA; (E) HNTs–KH560–CNF starch–PVA; (F) HNTs–KH570–CNF starch–PVA.

The TS of the starch–PVA membranes were reinforced by the nanoparticles. As shown in Figure 2a, the introduction of HNTs and CNF increased, respectively, 27.53% and 48.31% in TS compared to pristine starch–PVA due to the hydrogen bonding interaction between both of them and starch–PVA. It was found that the introduction of HNTs–CNF exhibited a further increment of 50% in TS compared with control starch–PVA owing to the hydrogen bonding interaction and the cross-linking network. Moreover, compared to the pure starch–PVA, the TS value reached 8.55 MPa and the maximum of 8.99 MPa after adding the HNTs–KH560–CNF and HNTs–KH570–CNF, realizing increments of 60.11% and 68.35%, respectively, which indicated the effects of the generated silicon hydroxyl group from the hydrolysis of silane agents, reacting with the hydroxyl group on the HNTs’ surface [37]. The epoxy group on KH560 was opened and grafted with the double bond on PVA. The double bond of KH570 can also graft with the double bond on PVA, so as to closely connect HNTs, CNF and the starch–PVA membrane. In addition, the contribution of HNTs to mechanical properties, including TS and elongation at break (EB) of starch–PVA composite membranes, is shown in Figure 2. As shown in Figure 2b, the EB increased from 103.6% to 201.3%, which proved the flexibility of the composites was enhanced because the addition of the nanoparticles made the cross-linking structure the in membranes tighter and firmer.

### 3.3. Water Resistance Properties of Starch–PVA Composite Membrane 

**Figure 3:**
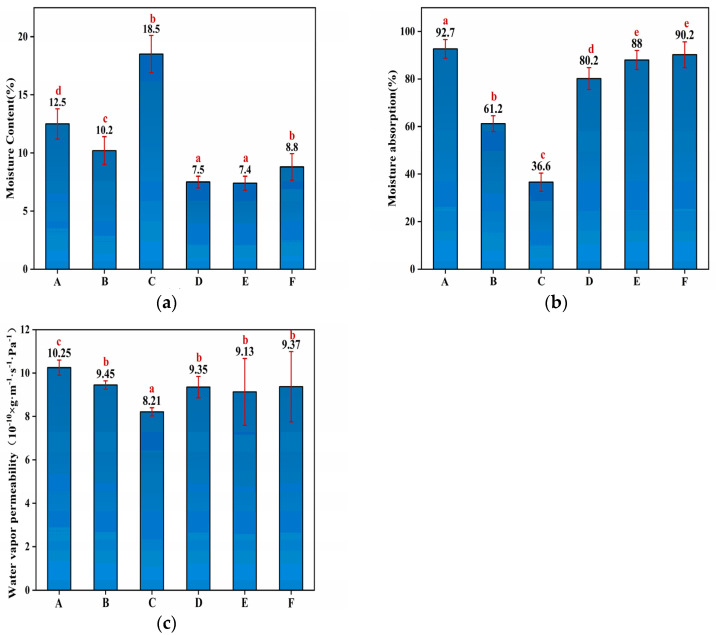
The (**a**) MC; (**b**) MA; (**c**) WVP of composite membrane samples of (A) starch–PVA; (B) HNTs/starch–PVA; (C) CNF/starch–PVA; (D) HNTs–CNF/starch–PVA; (E) HNTs–KH560–CNF starch–PVA; (F) HNTs–KH570–CNF starch–PVA.

The water resistance characteristics of membranes were characterized by measuring their respective moisture uptake at various humidity levels. The MC, MA and WVP values of the HNTs/starch–PVA nanocomposites are shown in Figure 3. The MC values of the different HNTs/starch–PVA nanocomposites were nearly identical, except for the incorporation of the CNF, which significantly enhanced the MC due to the large specific surface area and the active hydroxyl groups of CNF [38]. The incorporation of the HNTs, CNF, HNTs–CNF, HNTs–KH570–CNF and HNTs–KH560–CNF decreased the MA values of membranes from 92.7% in the control membrane to 61.2%, 36.6%, 80.2%, 88% and 90.2%, respectively. The significant decrease of MA in HNTs/starch–PVA may be attributed to the nanotube inner immobilization and active interfacial bonding to the starch–PVA matrix. The surface properties of materials were related to their tendency of absorbing water in the environment, which was of great significance for the application of biopolymers.

The WVP was also used to further investigate water resistance properties of HNTs/starch–PVA composite membranes [39]. The WVP values of different HNTs/starch–PVA composite membranes were nearly identical. It could be explained that as water evaporated from the material, the polar groups were more easily exposed to the edges of the membrane. The KH560 and KH570 in the polymer reacted with HNTs and CNF to form less penetrable outermost surfaces that partially prevent moisture invasion.

### 3.4. Micromorphological Analysis of Composite Membranes

**Figure 4:**
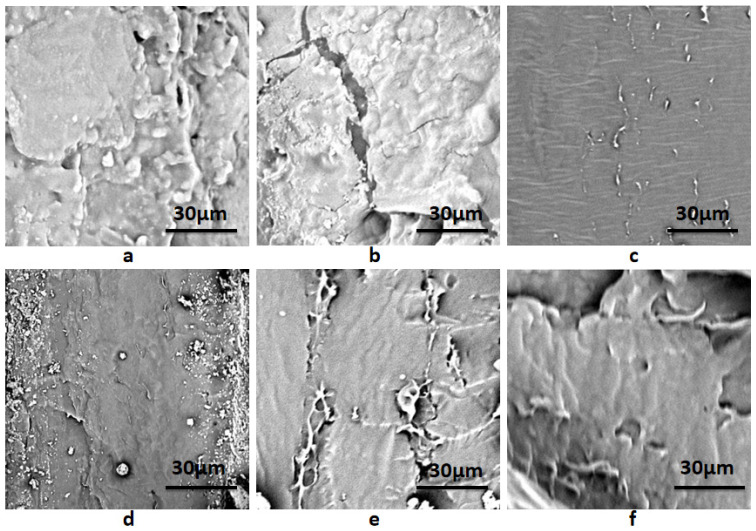
SEM images of composite membrane samples of (**a**) starch–PVA; (**b**) HNTs/starch–PVA; (**c**) CNF/starch–PVA; (**d**) HNTs–CNF/starch–PVA; (**e**) HNTs–KH560–CNF starch–PVA; (**f**) HNTs–KH570–CNF starch–PVA.

The SEM photographs of composite membranes are shown in Figure 4. It could be seen that the fracture surface of the starch–PVA composite was relatively smooth and homogeneous. As shown in Figure 4a, starch–PVA chains self-aggregate and form a dense network. As opposed to the starch–PVA composite in Figure 4a, the fracture cleavages and coarse structure in the HNTs/starch–PVA nanocomposite were examined, mainly due to the difficulty in obtaining well-dispersed HNTs, which further affected the morphological properties. However, it was found that in Figure 4c, the fracture surface was more uniform and smoother than the starch–PVA composite, indicating that the CNF was dispersed evenly, which was attributed to its good dispersion. It was observed that in Figure 4d, the HNTs–CNF/starch–PVA sample contained multiple interfacial bonding sites to combine with the peptide chains. Cano et al. proposed that the cassava starch–PVA membranes have higher elasticity values due to their more compact structure [40]. This change guaranteed the high mechanical properties of the starch–PVA composite. Moreover, as presented in Figure 4e–f, as the starch polymer changed from brittle fracture to ductile fracture, some fiber morphologies and some layered structures appeared on the fracture surface of the modified nanocomposites, which could make nanoparticles better dispersed in starch–PVA composites. Starch–PVA composites could reduce the stress in the tensile fracture process and prevented the entanglement between starch polyvinyl alcohol chains, so as to promote the effective two-phase bonding in the nanoparticle polymer interface and increase TS.

### 3.5. FTIR Analysis of Composite Membranes

**Figure 5:**
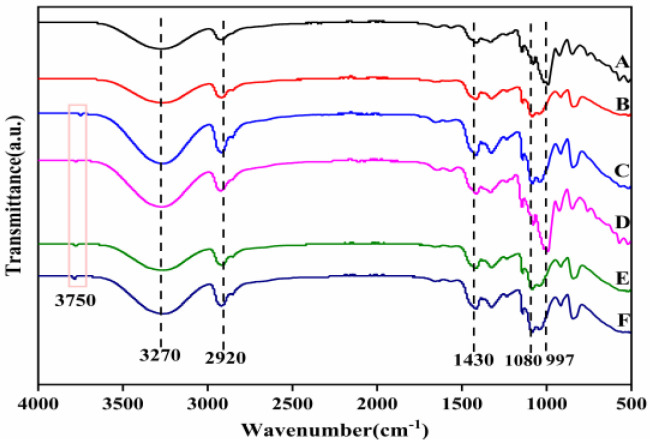
The FTIR spectra of composite membrane samples (A) starch–PVA; (B) HNTs/starch–PVA; (C) CNF/starch–PVA; (D)HNTs–CNF/starch–PVA; (E) HNTs–KH560–CNF starch–PVA; (F) HNTs–KH570–CNF starch–PVA.

FTIR studies were performed in order to explore changes in the functional groups of the composites prepared, as shown in Figure 5. It was observed that the spectral peak positions of all films were similar. The absorption peak at the 3270 cm^−1^ wave number was an O−H stretching vibration, which was caused by the strong intramolecular and intermolecular hydrogen bonding at 2920 cm^−1^ (C–H stretching), 650 cm^−1^ (O–H symmetric bending stretching) and 997 cm^−1^ (C–O stretching). This indicated that there were –OH and –COOH on the surface of starch–PVA nanocomposites, which was consistent with other research results [41,42]. Based on the above results, it could be concluded that –OH and –COOH existed on the surface of CNF–HNTs, which could form hydrogen bonds with starch rather than chemical crosslinking. After adding HNTs, due to the tensile vibration of Al-OH groups on the surface of HNTs, a weak absorption peak appeared at the 3750 cm^−1^ wave number. The absorption peak corresponding to the bending vibration of Si–O bond appeared at the wave number of 1056 cm^−1^, indicating the interaction between them.

### 3.6. XRD Analysis of Composite Membrane

**Figure 6:**
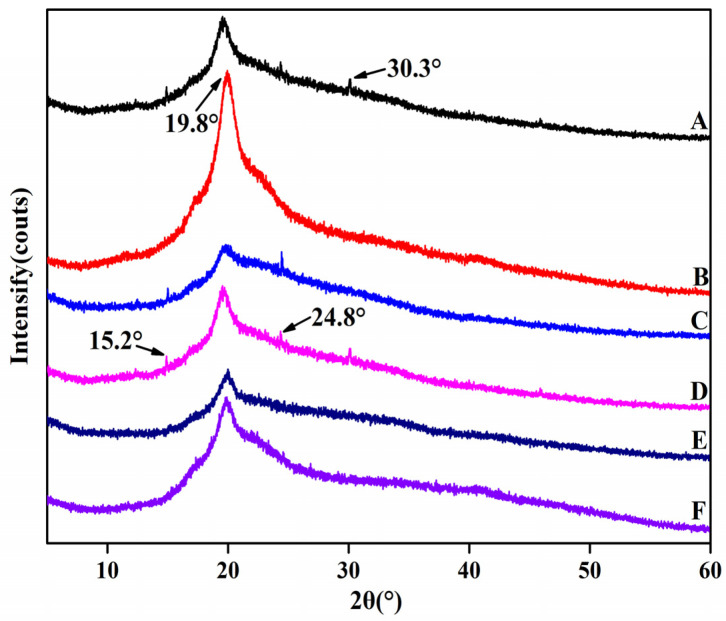
XRD patterns of composite membrane samples (A) starch–PVA; (B) HNTs/starch–PVA; (C) CNF/starch–PVA; (D) HNTs–CNF/starch–PVA; (E) HNTs–KH560–CNF starch–PVA; (F) HNTs–KH570–CNF starch–PVA.

The XRD measurement was employed to demonstrate the structural conformations of the HNTs/starch–PVA composite membranes (Figure 6). Compared to the starch–PVA membrane, new peaks appeared in HNTs/starch–PVA composite membranes at 2θ values of 15.2°, 19.8°, 24.8° and 30.3°, which referred to the characteristic peaks of the HNTs. With the incorporation of HNTs, the intensity of the diffraction peaks at 8.4° decreased, possibly due to the discontinuity of nanotubes filling the starch–PVA matrix [43]. The crystallinity of starch–PVA and HNTs/starch–PVA composite membranes was calculated according to Formula (1), as shown in Table 2. It could be seen that the degree of crystallinity compared to that of the pristine starch–PVA composite increased upon the addition of the HNTs, which was probably the result of the blending of the highly crystalline nanotubes and the starch–PVA chains partially penetrating into the nanotubes with an orderly alignment. As shown in Figure 6, the XRD pattern of the CNF/starch–PVA nanocomposite with CNF added showed that only a broad diffraction peak showed up near 2θ ≈ 19.8°, compared to the XRD pattern of the starch–PVA membrane. In comparison with the pristine starch–PVA composite, CNF/starch–PVA nanocomposite demonstrated that only a broad diffraction peak showed up near 2θ ≈ 19.8° and the remaining three weak diffraction peaks disappeared, which indicates that the CNF and starch–PVA were well combined. However, due to the good dispersion of CNF, the order of molecular chain was destroyed, resulting in the decrease of crystallinity of starch–PVA composite membranes. Compared to the starch–PVA composite, the HNTs/starch–PVA composite membrane showed that the weak diffraction peaks existing around 2θ ≈ 30.2° disappeared and the crystallinity increased significantly, which was a results of the effects of the HNTs and starch–PVA chains. The formation of hydrogen bonds between these substances also promoted the formation of crystals. The HNTs had a strong hydrogen-bonding orientation, which was conducive to the enhancement of the degree of intermolecular ordering, and thus the crystallinity. The addition of the HNTs–CNF nanoparticle system also formed a cross-linked network structure without destroying the crystalline structure of the starch–PVA membrane, and improved the order and compactness of the molecular structure, thereby improving the crystallinity further [44,45].

The XRD pattern of CNF–HNT nanoparticles modified by silane coupling agent added to the starch–PVA was compared with the XRD pattern of pristine starch–PVA. The result showed that with the introduction of nanoparticles modified by silane coupling agent, the other three weak diffraction peaks disappeared and the crystallinity of nanocomposites improved. This was because the SiO_2_ (epoxy group contained in KH560 and the double bond of KH570 itself) in the silane coupling agent was all hydrogen bonded in the structure of the starch–PVA and cross-links, forming a dense structure.

### 3.7. Thermal Performance of the HNTs/Starch–PVA Composite Membrane

**Figure 7:**
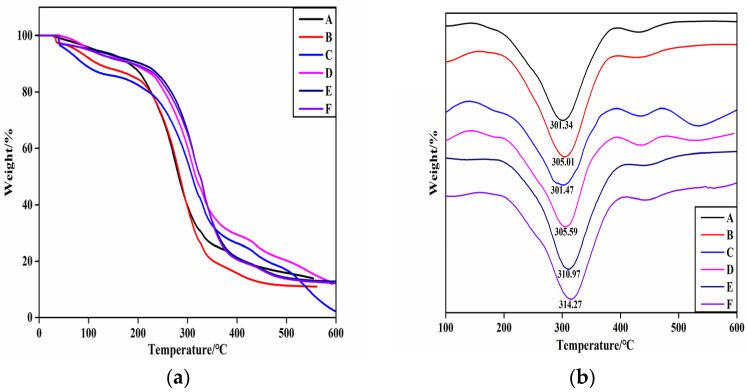
The (**a**) TG; (**b**) DTG curves of composite membrane samples (A) starch–PVA; (B) HNTs/starch–PVA; (C) CNF/starch–PVA; (D) HNTs–CNF/starch–PVA; (E) HNTs–KH560–CNF starch–PVA; (F) HNTs–KH570–CNF starch–PVA.

The weight loss traces and derivative TG (DTG) curves of the HNTs/starch–PVA composite membrane, which presented similar thermal behavior, are shown in Figure 7.

There were two main degradation stages exclusive to the dehydration reaction (before 120 °C) and glycerol degradation from 120 °C to 200 °C. The significant degradation rate of membranes appeared between 285 °C and 450 °C, resulting from the degradation of the starch–PVA matrix [46]. During the initial stage, glycerol degradation was slower in the HNTs/starch–PVA composite membrane. A possible explanation for this was that the nanotubes imped the escape of volatile products through barrier and entrapment effects. The dispersion of the HNTs was also likely to complicate the pathways, hindering decomposition of small molecules. However, we hypothesized that the HNTs–CNF, HNTs–KH560–CNF and HNTs–KH570–CNF samples dispersed more uniformly than others and had a favorable interfacial combination with the starch–PVA matrix. Therefore, it could be concluded that the incorporation of HNTs did not have much influence on the thermal properties of starch–PVA nanocomposites. The main thermal decomposition stage of HNTs was in the range of 461.2 °C–527.3 °C. The peak of the DTG curve appeared at 520 °C, which was attributed to the dihydroxylation of Al–OH groups in the remaining structure. In the modified HNT sample, the degradation of the framework was partially because of the evaporation and silane grafting of the two bonded water molecules, resulting in a weight loss at 200.0 °C to 461.2 °C. This stage progress slowly and partially overlapped with other phases. Sun et al. made similar observations. After 527.3 °C, a further degradation stage occurred, which may be related to the formation of SiO_2_ residue during thermal degradation of KH560 and KH570. This was probably because the weight loss of the modified HNTs included some surface-modified silane-coupled oligomers and side group thermal decomposition. Furthermore, the thermal stabilization of the biopolymer agreed with the good dispersion of the fillers into the polymeric matrix [47].

As can be seen from the figure comparing other composites with the starch–PVA composite, the maximum weight loss rate of other composite membranes was higher, confirming the higher thermal stability. This was possibly because the starch–PVA matrix was tightly cross-linked with the nanoparticles, meaning that decomposition could not easily happen and the thermal stability of the composites was improved. Furthermore, the decomposition rate of the DTG curve of nanocomposites before 200 °C was almost zero, which showed that the addition of nanoparticles with silane coupling agent slightly improved the thermal stability and improved the heat resistance of the composites. The decomposition temperature increased from 301 °C to 314 °C, which indicated that the silane coupling agent-modified specimens had formed chemical cross-linking action with the starch and PVA molecule. Overall, the results indicated that KH560 and KH570 were likely to have successfully chemically modified CNF/HNTs, and the heat resistance of the starch–PVA composite membranes had been significantly improved [34].

### 3.8. DSC Studies of the HNTs/Starch–PVA Composite Membrane 

Typical DSC dates are summarized in Table 3. It was found that both T_g_ and T_m_ of PVA increased in the presence of starch, presumably because of the stiffening effect of the hydrogen bonding interaction with starch [48]. Incorporation with HNTs and CNF caused an increase of both T_g_ and T_m_. However, the higher T_g_ and T_m_ suggested that the average crystal size in nanocomposites was larger, which was the result of the strengthening of the PVA–starch bonding under the presence of the nanofillers [40]. Moreover, the heat of melting of starch–PVA nanocomposites decreased significantly without the addition of the silane coupling agent, which indicated the decrease of the percentage of crystallinity of PVA in the nanoparticle/polymer systems. However, incorporation of HNTs in the absence of the silane coupling agent did not cause further significant changes in crystallinity of the starch–PVA composite membrane.

## 4. Conclusions

In this work, the facile surface modification of the internal/external HNT surfaces was performed using functional silane grafting. CNF was used to surround both sides of the nanotube, while epoxy group and double bond were, respectively, fixed on CNF by silane grafting KH560 and KH570. The active and functional surfaces of HNTs–KH560–CNF and HNTs–KH570–CNF showed superb interfacial adhesions with the penetrated and entangled starch–PVA chains. The results showed that the TS of the HNTs–KH560–CNF/starch–PVA improved from 5.34 MPa to 8.50MPa and the TS of HNTs–KH570–CNF/starch–PVA improved from 5.34 MPa to 8.90 MPa. The functional surfaces featuring dense reaction sites further enhanced the water resistance of the composites supported by the MC, MA and WVP results. The introduction of chemical bonds formed associations and a compact network structure, which increased the thermal stability and the crystallinity of the starch–PVA composite membrane. In conclusion, in this study, starch–PVA biodegradable composite membranes were fabricated and their properties were characterized. HNTs as reinforcement improved the physical properties of the starch–PVA membrane. The addition of CNF improved the dispersion of HNTs and caused it to be better dispersed in the membrane. The addition of silane coupling agent grafted CNF and HNTs together, which improved the thermal stability and film density. The practicability of the starch–PVA membrane was greatly improved after being reinforced by nanoparticles. It is expected to become a new degradable packaging material.

## Figures and Tables

**Table 1:** The detailed code and formulations of all the composite membranes.

Code	5% Starch Solutions	5% PVA Solutions	1% Nanofillers Solution
A	20 g	10 g	-
B	20 g	10 g	7.5 g HNTs
C	20 g	10 g	7.5 g CNF
D	20 g	10 g	7.5 g HNTs-CNF
E	20 g	10 g	7.5 g HNTs-KH560-CNF
F	20 g	10 g	7.5 g HNTs-KH570-CNF

**Table 2:** Crystal properties of membrane by X-ray diffraction.

Code	Angle 2θ/°	d/Å	Degree of Crystallinity
A	19.750°	4.4915	39.44%
B	19.980°	4.4403	38.18%
C	19.860°	4.4668	43.86%
D	19.790°	4.4825	41.45%
E	19.990°	4.4381	47.93%
F	19.890°	4.4602	40.37%

**Table 3:** DSC studies of starch–PVA membranes.

Code	Cooling				Heating			
	Tc1 (°C)	Tc2 (°C)	Hc (W/g)	Tg (°C)	Tm1 (°C)	Tm2 (°C)	Hm (W/g)	X (%)
A	161	192.7	11.48	100.8	210.1	213.6	8.742	60.08
B	156.7	193.5	15.02	88.7	205.9	216.8	6.308	43.35
C	170.2	193.9	14.94	111.5	210.1	220.9	6.03	41.44
D	165.4	192.8	19.57	104.9	214.6	217.6	7.91	54.36
E	163.2	192.8	15.77	107.9	209.9	217.3	12.89	88.59
F	170.6	194.8	15.32	105.1	208.4	220.3	10.18	69.96

## References

[1] Pacheco M.S., Barbieri D., da Silva C.F., de Moraes M.A. (2021). A review on orally disintegrating membranes (ODFs) made from natural polymers such as pullulan, maltodextrin, starch, and others. Int. J. Biol. Macromol..

[2] Prabhu T.N., Prashantha K. (2018). A review on present status and future challenges of starch based polymer membranes and their composites in food packaging applications. Polym. Compos..

[3] Wilfer P.B., Giridaran G., Jeevahan J.J., Joseph G.B., Kumar G.S., Thykattuserry N.J. (2021). Effect of starch type on the membrane properties of native starch based edible membranes. Mater. Today Proc..

[4] Sadegh-Hassani F., Mohammadi Nafchi A. (2014). Preparation and characterization of bionanocomposite membranes based on potato starch/halloysite nanoclay. Int. J. Biol. Macromol..

[5] Van Long N.N., Joly C., Dantigny P. (2016). Active packaging with antifungal activities. Int. J. Food Microbiol..

[6] Zhu Z.F., Wang M., Li W. (2015). Starch maleation and sulfosuccinylation to alleviate the intrinsic drawback of brittleness of cornstarch membrane for warp sizing. Fibers Polym..

[7] Riyajan S.A., Chantawee K. (2020). Cassava starch composite based membranes for encapsulated neem: Effect of carboxylated styrene-butadiene rubber coating. Food Packag. Shelf Life.

[8] Sharma V., Kaur M., Sandhu K.S., Nain V., Janghu S. (2021). Physicochemical and rheological properties of cross-linked litchi kernel starch and its application in development of bio-membranes. Starch-Stärke.

[9] Ordonez R., Atares L., Chiralt A. (2021). Physicochemical and antimicrobial properties of cassava starch membranes with ferulic or cinnamic acid. LWT-Food Sci. Technol..

[10] Yan X.X., Diao M.X., Yu Y.L., Gao F., Wang E.L., Wang Z.Z., Zhang T.H. (2021). Influence of esterification and ultrasound treatment on formation and properties of starch nanoparticles and their impact as a filler on chitosan based membranes characteristics. Int. J. Biol. Macromol..

[11] Liu P.F., Gao W., Zhang X.L., Wang B., Zou F.X., Yu B., Lu L., Fang Y.S., Wu Z.Z., Yuan C. (2021). Effects of ultrasonication on the properties of maize starch/stearic acid/sodium carboxymethyl cellulose composite membrane. Ultrason. Sonochem..

[12] Sani I.K., Geshlaghi S.P., Pirsa S., Asdagh A. (2021). Composite membrane based on potato starch/apple peel pectin/ZrO_2_ nanoparticles/microencapsulated *Zataria multiflora* essential oil; Investigation of physicochemical properties and use in quail meat packaging. Food Hydrocoll..

[13] Wu S.L., Wang W.T., Zhang R., Zhai X.S., Hou H.X. (2021). Preparation and characterization of biodegradable trilayer membranes based on starch and polyester. Int. J. Biol. Macromol..

[14] Trinh B.M., Chang C.C., Mekonnen T.H. (2021). Facile fabrication of thermoplastic starch/poly (lactic acid) multilayer membranes with superior gas and moisture barrier properties. Polymer.

[15] Hernandez-Garcia E., Vargas M., Chiralt A. (2021). Thermoprocessed starch-polyester bilayer membranes as affected by the addition of gellan or xanthan gum. Food Hydrocoll..

[16] Tang H.L., Xiong H.G., Tang S.W., Zou P. (2009). A starch-based biodegradable membrane modified by nano silicon dioxide. J. Appl. Polym. Sci..

[17] Mohan T.P., Kanny K. (2016). Thermoforming studies of corn starch-derived biopolymer membrane filled with nanoclays. J. Plast. Membr. Sheeting.

[18] Belibi P.C., Daou T.J., Ndjaka J.M.B., Michelin L., Brendlé J., Nsom B., Durand B. (2013). Tensile and water barrier properties of cassava starch composite membranes reinforced by synthetic zeolite and beidellite. J. Food Eng..

[19] Gou Z.Q. (2021). The synergistic reinforcing effects of nano-diamond and chitosan-grafted MWNTs in starch membranes. Starch-Stärke.

[20] Devi N., Dutta J. (2019). Development and in vitro characterization of chitosan/starch/halloysite nanotubes ternary nanocomposite membranes. Int. J. Biol. Macromol..

[21] Noshirvani N., Ghanbarzadeh B., Fasihi B., Almasi H. (2016). Starch-PVA nanocomposite membrane incorporated with cellulose nanocrystals and MMT: A comparative study. Int. J. Food Eng..

[22] Ortega F., Arce V.B., Garcia M.A. (2021). Nanocomposite starch-based membranes containing silver nanoparticles synthesized with lemon juice as reducing and stabilizing agent. Carbohydr. Polym..

[23] Chen X.H., Zhou L.Y., Pan X.M., Hu J.H., Hu Y.X., Wei S.S. (2016). Effect of different compatibilizers on the mechanical and thermal properties of starch/polypropylene blends. J. Appl. Polym. Sci..

[24] Yang L., Xie M.Z., Fang J.X., Zhang T.Y., Wang X.M., Chen L.P. (2022). Effect of additives on properties of cross-linked carboxymethyl starch/polyvinyl alcohol composite membranes. J. Appl. Polym. Sci..

[25] Patil S., Bharimalla A.K., Mahapatra A., Dhakane-Lad J., Arputharaj A., Kumar M., Raja A.S.M., Kambli N. (2021). Effect of polymer blending on mechanical and barrier properties of starch-polyvinyl alcohol based biodegradable composite membranes. Food Biosci..

[26] Abdullah Z.W., Dong Y. (2019). Biodegradable and water resistant poly(vinyl) alcohol (PVA)/starch (ST)/glycerol (GL)/halloysite nanotube (HNT) nanocomposite membranes for sustainable food packaging. Front. Mater..

[27] Sarifuddin N., Ismail H., Ahmad Z. (2014). Influence of halloysite nanotubes hybridized with kenaf core fibers on the physical and mechanical properties of low density polyethylene/thermoplastic sago starch blends. Polym.-Plast. Technol. Eng..

[28] He Y.Q., Kong W.N., Wang W.C., Liu T.L., Liu Y., Gong Q.J., Gao J.P. (2012). Modified natural halloysite/potato starch composite membranes. Carbohydr. Polym..

[29] Lazzara G., Cavallaro G., Panchal A., Fakhrullin R., Stavitskaya A., Vinokurov V., Lvov Y. (2018). An assembly of organic-inorganic composites using halloysite clay nanotubes. Curr. Opin. Colloid Interface Sci..

[30] Soni B., Hassan E.B., Mahmoud B. (2015). Chemical isolation and characterization of different cellulose nanofibers from cotton stalks. Carbohydr. Polym..

[31] De Almeida T.S., Barretti B.R.V., Ito V.C., Malucelli L., Da Silva Carvalho Filho M.A., Demiate I.M., Pinheiro L.A., Lacerda L.G. (2020). Thermal, morphological, and mechanical properties of regular and waxy maize starch membranes reinforced with cellulose nanofibers (CNF). Mater. Res.-Ibero-Am. J. Mater..

[32] Qin Z., Mo L., Liao M., He H., Sun J. (2019). Preparation and characterization of soy protein isolate-based nanocomposite membranes with cellulose nanofibers and nano-silica via silane grafting. Polymers.

[33] Fazeli M., Keley M., Biazar E. (2018). Preparation and characterization of starch-based composite membranes reinforced by cellulose nanofibers. Int. J. Biol. Macromol..

[34] Jose J., Al-Harthi M.A., AlMa’adeed M.A.A., Dakua J.B., De S.K. (2015). Effect of graphene loading on thermomechanical properties of poly(vinyl alcohol)/starch blend. J. Appl. Polym. Sci..

[35] Zhang S.F., Xia C.L., Dong Y.M., Yan Y.T., Li J.Z., Shi S.Q., Cai L.P. (2016). Soy protein isolate-based membranes reinforced by surface modified cellulose nanocrystal. Ind. Crop. Prod..

[36] Gutiérrez T.J., Tapia M.S., Pérez E., Famá L. (2015). Structural and mechanical properties of edible membranes made from native and modified cush-cush yam and cassava starch. Food Hydrocoll..

[37] Panaitescu D.M., Frone A.N., Ghiurea M., Chiulan I. (2015). Influence of storage conditions on starch/PVA membranes containing cellulose nanofibers. Ind. Crop. Prod..

[38] Aguirre-Loredo R.Y., Rodríguez-Hernández A.I., Morales-Sánchez E., Gómez-Aldapa C.A., Velazquez G. (2016). Effect of equilibrium moisture content on barrier, mechanical and thermal properties of chitosan membranes. Food Chem..

[39] Khalil H.P.S.A., Yap S.W., Tye Y.Y., Tahir P.M., Rizal S., Fazita M.R.N. (2018). Effects of corn starch and *Kappaphycus alvarezii* seaweed blend concentration on the optical, mechanical, and water vapor barrier properties of composite membranes. Bioresources.

[40] Cano A., Fortunati E., Cháfer M., González-Martínez C., Chiralt A., Kenny J.M. (2015). Effect of cellulose nanocrystals on the properties of pea starch–poly(vinyl alcohol) blend membranes. J. Mater. Sci..

[41] Hu D.Y., Wang L.J. (2016). Physical and antibacterial properties of polyvinyl alcohol membranes reinforced with quaternized cellulose. J. Appl. Polym. Sci..

[42] Kang H.J., Liu X.R., Zhang S.F., Li J.Z. (2017). Functionalization of halloysite nanotubes (HNTs) via mussel-inspired surface modification and silane grafting for HNTs/soy protein isolate nanocomposite membrane preparation. RSC Adv..

[43] Meira S.M.M., Zehetmeyer G., Scheibel J.M., Werner J.O., Brandelli A. (2016). Starch-halloysite nanocomposites containing nisin: Characterization and inhibition of *Listeria* monocytogenes in soft cheese. LWT-Food Sci. Technol..

[44] Mansour G., Zoumaki M., Marinopoulou A., Raphaelides S.N., Tzetzis D., Zoumakis N. (2020). Investigation on the effects of glycerol and clay contents on the structure and mechanical properties of maize starch nanocomposite membranes. Starch-Stärke.

[45] Jayakumar A., Heera K.V., Sumi T.S., Joseph M., Mathew S., Praveen G., Nair I.C., Radhakrishnan E.K. (2019). Starch-PVA composite membranes with zinc-oxide nanoparticles and phytochemicals as intelligent pH sensing wraps for food packaging application. Int. J. Biol. Macromol..

[46] Ali M.A.S.S., Jimat D.N., Nawawi W.M.F.W., Sulaiman S. (2021). Antibacterial, mechanical and thermal properties of PVA/starch composite membrane reinforced with cellulose nanofiber of sugarcane bagasse. Arab. J. Sci. Eng..

[47] Cavallaro G., Lazzara G., Milioto S. (2013). Sustainable nanocomposites based on halloysite nanotubes and pectin/polyethylene glycol blend. Polym. Degrad. Stab..

[48] Kumar A., Chouhan D.K., Alegaonkar P.S., Patro T.U. (2016). Graphene-like nanocarbon: An effective nanofiller for improving the mechanical and thermal properties of polymer at low weight fractions. Compos. Sci. Technol..

